# Comparison of retrograde ureterorenoscopy (URS) and percutaneous anterograde ureteroscopy for removal of impacted upper ureteral stones >10mm in the elderly population

**DOI:** 10.1590/S1677-5538.IBJU.2019.0638

**Published:** 2020-11-18

**Authors:** Mehmet İlker Gökce, Çağri Akpinar, Khaled Obaid, Evren Süer, Ömer Gülpinar, Yaşar Bedük

**Affiliations:** 1 Ankara University School of Medicine Department of Urology Ankara Turkey Department of Urology, Ankara University School of Medicine, Ankara, Turkey

**Keywords:** Ureteroscopy, Nephrostomy, Percutaneous, Housing for the Elderly, Ureteral Obstruction

## Abstract

**Purpose:**

We aimed to compare the success and complication rates of the anterograde and retrograde Ureterorenoscopy (URS) for impacted upper ureteral stones in patients > 65 years of age.

**Materials and Methods:**

Data of 146 patients >65 years of age and underwent anterograde URS (n=68) in supine position or retrograde URS (n=78) for upper ureteral impacted stones>10 mm between January 2014 and September 2018 were collected prospectively. The groups were compared for success and complication rates, duration of operation, hospital stay, and ancillary procedures.

**Results:**

Anterograde and retrograde URS groups were similar for demographic and stone related characteristics. The success rate of the anterograde URS group was significantly higher than the retrograde URS group (97.1% vs. 78.2%, p=0.0007). The complication rates were similar for the two groups (p=0.86). Clavien grade I and II complications were observed in 3 patients in each group. The mean hemoglobin drop was 0.5 g/dL in the anterograde URS group and blood transfusion was not performed in any of the patients. The mean duration of operation was 41.2±12.5 minutes in the mini-PNL group and 59.6±15.1 minutes in the RIRS group and the difference was statistically significant (p=0.02). The median duration of hospitalization was 1 day for both groups.

**Conclusions:**

Performing anterograde URS in supine position provided better success rates and similar complication rates compared to retrograde URS. Based on these results anterograde URS shall be considered as one of the primary treatment options for management of impacted upper ureteral stones in the elderly population.

## INTRODUCTION

The main goal of an endourology procedure is to achieve stone free status with minimal morbidity. Ureterorenoscopy (URS) and shock wave lithotripsy (SWL) are the main treatment modalities for upper ureteral stones and the most recent EAU and AUA guidelines recommend URS as the primary modality for stones >10mm (1, 2).

In case the stone is located at the same level of ureter for over 2 months period, it is defined as an impacted stone. In such case URS is the main treatment option (3, 4). Percutaneous anterograde URS is recommended by the EAU guidelines when the collecting system is dilated or when the ureter is inconvenient for retrograde manipulation (2).

Retrograde and anterograde URS for upper ureteral impacted stones have been compared in a number of studies (5–10) and the results of these studies were included in a recent meta-analysis (11). All of the prospective randomized studies were performed in prone position and the authors concluded that anterograde URS should be the primary treatment option for an impacted upper ureteral stone due to its higher success rate and similar complication rates (11).

Management of stones in the elderly population is particularly important due to increased rate of comorbidities and vulnerability of the kidneys to obstruction. Therefore, a study comparing retrograde URS and anterograde URS for impacted upper ureteral stones in the elderly population is of importance. None of the previously published studies focus on the elderly population and in this prospective comparative study we aimed to compare the success and complication rates of the anterograde and retrograde URS in patients >65 years of age.

## MATERIALS AND METHODS

Data of 146 patients >65 years of age that underwent surgery for upper ureteral impacted stones between January 2014 and September 2018 were collected prospectively. The patients were grouped as anterograde URS (n=68) and retrograde URS (n=78). Patients were informed about both anterograde or retrograde URS with respect to the success and complication rates and also the postoperative course. The type of the procedure was selected based on patient's preferences without any randomization. All of the patient informing process and the operations were performed by a single experienced surgeon. Patients with stones 10-25mm, requiring active intervention, and with no bleeding diathesis were included. All of the patients underwent non-contrast CT scan preoperatively and the stone size was reported as the longest diameter of the stone in CT scan. Radio-opacity of the stones were determined from the scout films of the CT scans. Antibiotic therapy to establish a sterile urine culture prior to surgery was given to all patients with positive urine cultures. The study complied with the guidelines for human studies. All subjects have given their informed consent prior to study and the study was approved by Ethical Committee of our institution.

### Surgical methods

Retrograde URS: Patients were positioned in lithotomy position and initially URS was performed with 6.5Fr semirigid ureteroscope (Karl-Storz, Tuttlingen, Germany) to check the ureter for any pathology and caliber. A guidewire was placed in the collecting system and a 9.5/11.5Fr ureteral access sheath (Cook, Flexor®, Bloomington, IN, USA) was placed if the ureter was compliant. Flexible URS (Flex X2, Karl-Storz, Tuttlingen, Germany) was advanced through the access sheath and laser lithotripsy in low energy and high frequency setting (0.4-0.6 J and 20Hz) was performed. In case of residual fragments within the kidney, popcorn lithotripsy with 1.0-1.5 J and 8-10Hz was also performed to decrease the size of the fragments.

Fragments were extracted with a nitinol basket at the end of the procedure and a JJ stent was placed in all of the cases.

Anterograde URS: Patients were placed in Galdakao modified supine Valdivia position. Initially URS was performed with 6.5Fr semirigid ureteroscope (Karl-Storz, Tuttlingen, Germany) in order to evaluate if there was any ureteral stricture or any stone fragment migrated to distal parts of the ureter. A 6Fr ureteral catheter was placed and retrograde pyelogram was performed. Percutaneous access was performed through a middle or upper calyx to access to the ureter with a favorable angle. The MIP-M kit (Karl Storz, Tuttlingen, Germany) was used to create percutaneous tract with 15Fr metallic dilation and 16Fr metallic sheath was placed 12Fr nephroscope was introduced through the upper ureter and laser lithotripsy was performed with fragmentation settings (1.5-2.0 J and 10Hz). The fragments were extracted with the vacuum cleaner effect and retrograde irrigation through the ureteral catheter was performed to assist extraction of the fragments when needed. If all of the stone fragments could not be reached with the rigid nephroscope, flexible URS was used through the percutaneous sheath. A JJ stent was placed in all cases and nephrostomy tube was not placed in any of the cases.

Hemoglobin levels were measured preoperatively in all of the cases and in the anterograde URS group, postoperative hemoglobin levels were also measured and amount of hemoglobin drop was recorded. The JJ stent was extracted 14 days after surgery and imaging was performed with ultrasound for radiolucent stones and KUB for radio-opaque stones before stent extraction. In case of residual fragments greater than 2mm in longest diameter, low dose non-contrast CT was also performed. The success was defined as absence of residual fragments >2mm. Complications were recorded based on the Clavien-Dindo classification system (12). The primary end point of the study was success rate at the time of JJ stent extraction. The secondary end points were complication rates and rates of ancillary procedures.

### Statistical Analysis

Statistical analysis was performed with SPSS ver. 20.0 (IBM Corp. Released 2011. IBM SPSS Statistics for Windows®, Version 20.0. Armonk, NY: IBM Corp.) The normal distribution of the continuous variables was tested with the Kolmogorov-Smirnov test. Chi square test or Fisher's Exact test was used to compare categorical variables and Student t-test was applied to compare continuous variables. For statistical significance p value of 0.05 was accepted.

## RESULTS

The mean age of the population was 68.3±3.3. Anterograde and retrograde URS groups were similar for age, gender, body mass index, comorbidities, presence of severe hydronephrosis, and stone related characteristics. Prior to operation, a JJ stent was not placed in any of the patients. The results are summarized in Table-1.

**Table 1:** Demographic characteristics of the patients.

Parameters		Anterograde URS (n=68)	Retrograde URS (n=78)	P value
Age, mean±SD		67.8±3.3	68.7±3.5	0.47
**Gender, n(%)**				**0.73**
	Male	32 (47.1)	40 (51.3)	
	Female	36 (52.9)	38 (48.7)	
Stone size (mm), mean±SD		15.3±2.4	14.6±6.1	0.65
**Side, n(%)**				**0.42**
	Right	33 (48.5)	43 (55.1)	
	Left	35 (51.5)	35 (44.9)	
Stone density (HU), mean±SD		1025.2±275	1012±280	0.88
Body Mass index mean±SD		25.5±2.3	25.8±2.9	0.55
Presence of severe hydronephrosis		50 (73.5)	54 (69.2)	0.57
**ASA status, n(%)**				**0.35**
	ASA I	26 (38.2)	34 (43.6)	
	ASA II	39 (57.4)	36 (46.2)	
	ASA III	2 (2.9)	7 (8.9)	
	ASA IV or more	1 (1.5)	1 (1.3)	

**Figure 1:**
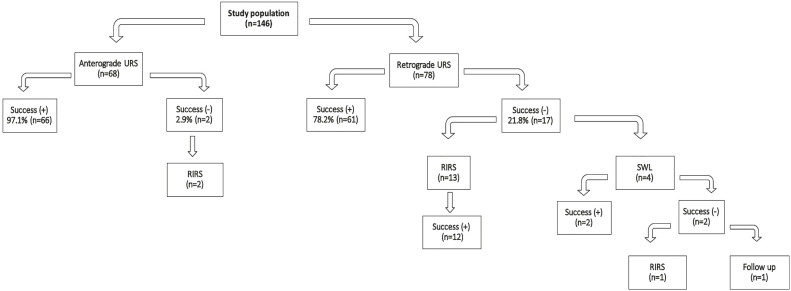
The results of the primary and the ancillary procedures in the anterograde and retrograde URS groups.

The success rate of the anterograde URS group was significantly higher than the retrograde URS group (97.1% vs. 78.2%, p=0.0007). During the surgery anterograde use of flexible URS was necessary in 5 of the 68 cases as it was not possible to reach all of the stones with the 12Fr nephroscope. In the anterograde URS group, only two of the 68 patients were found to have residual fragments with size of 4mm and 5mm. These fragments were extracted with retrograde intrarenal surgery (RIRS) during stent extraction and all of the patients were stone free at the end of the procedure. Ancillary procedures were not necessary in any of the patients thereafter. In the retrograde URS group, 17 (21.8%) patients were found to have residual stones. Four of these 17 patients underwent SWL and 2 of them established stone free status. In rest of the 13 patients RIRS was performed as ancillary procedure and in 12 of them stone free status was established. In one patient, a residual stone of 4mm was detected in the postoperative imaging and the patient was submitted to follow-up. The results of the primary and the ancillary procedures of both groups are summarized in Figure-1.

The complication rates were similar for the two groups (p=0.86). Clavien grade I and II complications were observed in 3 patients in each group. Clavien grade 3 or higher complications were not observed in any of the cases. The mean hemoglobin drop was 0.5±0.2g/dL in the anterograde URS group and blood transfusion was not performed in any of the patients. The mean duration of operation was 41.2±12.5 minutes in the anterograde URS group and 59.6±15.1 minutes in the RIRS group and the difference was statistically significant (p=0.02). The median duration of hospitalization was 1 day for both groups. The results are summarized in Table-2.

**Table 2:** Comparison of the two groups for the success, complications and hospitalization.

Parameters		Anterograde URS (n=68)	Retrograde URS (n=78)	P value
Success rate, n (%)		66 (97.1%)	61 (78.2%)	0.0007
**Complication rate, n (%)**				**0.86**
	Grade I	2 (2.9)	2 (2.6)	
	Grade II	1 (1.5)	1 (1.3)	
	Grade III or higher	–	–	
Hemoglobin drop (g/dL) mean± SD		0.5±0.2	Not measured	–
Duration of operation (minutes), mean±SD		41.2±12.5	59.6±15.1	0.02
Duration of hospitalization (days), median (range)		1 (1-2)	1 (1-3)	0.99

## DISCUSSION

The management of impacted ureteral stones in the elderly population is important and the endourologists should aim to balance the success and the complication rates of the surgical approaches. Anterograde and retrograde URS are the main surgical approaches for these cases and in this study we found out that performing anterograde URS in supine position has higher success rates and lower operative times than retrograde URS in the elderly population. The complication rates and the hospital stay of the two treatment modalities were similar.

Anterograde and retrograde URS have been compared in a randomized study by Sun et al. (7).

Different from our study, the mean age of the patients in this study was around 40 years and anterograde approach was performed in prone position. The success rate of the anterograde and retrograde approaches was 95.3% and 79.5% respectively and was similar to our findings. However, the mean hospital stay of the anterograde and retrograde groups were 6.3 days and 2.1 days respectively and these were quite longer compared to our cohort (7). In a more recent randomized study, Yang et al. compared retrograde URS with anterograde approach using a patented mini-PNL system (9). The stone free rates of the anterograde and retrograde approaches were 97.8% and 71.4% which were also similar to our results. Anterograde approach was performed in prone position and the duration of operation was 15-75 minutes and measured after the patient was positioned in prone position. However, the authors reported significantly greater amount of bleeding and higher treatment costs in the anterograde approach (9).

In another randomized study comparing retrograde URS and anterograde approach in prone position, the authors reported initial stone free rates of 41.4% and 93.3% for the two groups respectively (5). However, the stone free rates raised to 89.7% and 100% in the two groups during follow-up. The hospital stay of the anterograde URS group was 4.6 days and this is also higher compared to our cohort. We believe that this is probably due to placement of nephrostomy tubes and institutional policies for postoperative follow-up. We did not place a nephrostomy tube in any of the patients and median hospital stay of the group was 1 day. The authors also reported significant gross hematuria in 25 of the 29 patients in the anterograde URS group but blood transfusion was not required in any of the patients (5).

Zhang et al. compared anterograde URS (n=32) performed in supine position with retrograde approach (n=44) in a non-randomized prospective study (10). Different from our study, all of the patients in this study were pre-stented before retrograde URS. The authors reported stone free rate of 93.7% and 84.1% for the anterograde and retrograde URS groups respectively and the difference was not statistically significant. The groups were similar for postoperative complication rates but the mean operative time was significantly shorter in the anterograde URS group (49.3 minutes vs. 67.2 minutes, p <0.001) and mean hospital stay was significantly shorter in the retrograde URS group (4.2 days vs. 1.8 days). Based on these results the authors emphasized the similar stone free rates and faster recovery of the retrograde approach. However, in this study the number of patients was lower compared to our cohort and the power of the study was probably insufficient to detect the significance of difference between the stone free rates. Moreover, the mean hospital stay was >4 days like the previous studies but unlike our cohort (10).

The results of the studies mentioned above were included in a recent meta-analysis (11). The authors reported similar operative times for the anterograde and retrograde URS procedures but hospital stay was significantly longer in the anterograde URS group (mean difference of 3.14 days; 95%CI, 1.27 to 5.55). The overall stone free rate was also found to be significantly higher in the anterograde URS group (OR, 8.70; 95%CI, 3.23 to 23.45). The complication rates of the both groups were similar (11) except for hematuria, which was reported to be higher in only one study (5). Based on these findings the authors concluded that anterograde approach, with higher stone free rates and similar complication rates, should be the optimal treatment choice for management of impacted upper ureteral stones.

As the patient gets older, the renal functions and the general health status are more vulnerable and therefore, establishing a real stone free status by minimal morbidity is of upmost importance in the elderly population. SWL, being a minimally invasive alternative, is an important treatment option for ureteral stones. However, its efficiency in the elderly population has been questioned in a number of studies (13–15). URS with technical improvements provides excellent outcomes for upper ureteral stones.

The success of URS in the elderly population has been evaluated in a recent study and the authors reported 88% stone free rate which was slightly higher than our retrograde URS stone free rate. However, the authors did not provide any information on the stone impaction and only 29% of the stones were located in the ureteropelvic junction (16). In another recent study, Ozgor et al. compared success rate of RIRS and PNL for renal stones in a population of patients older than 60 years of age. The authors reported 81.7% and 77.6% stone free rates for the RIRS and PNL groups respectively. The authors concluded that both RIRS and PNL are effective treatment modalities in the elderly population but the latter is associated with higher complication rates and longer hospital stay (17). In our study, we found that performing anterograde URS in supine position was more successful than the retrograde URS and the safety profile of the two approaches were similar. We believe that, as the collecting system is dilated due to obstruction in the ureter and the calices were free of stones, renal access can easily be established. Besides, the anesthetic method did not differ for the anterograde and retrograde URS groups. As the patient was not placed in prone position, no additional complication risks were taken compared to retrograde URS performed in lithotomy position and the duration of surgery was also not prolonged.

The most important drawback of the current study is the non-randomized design. Although patients were enrolled prospectively, the non-randomized nature of the study results in significant risk of bias for patient selection. We also did not perform any power analysis but rather compared the outcomes of two surgical approaches performed in a given time period. But, the number of patients were sufficient to detect a significant difference between the two groups. Besides the postoperative imaging was not performed with non-contrast CT in all of the patients and we defined the success as no fragments >2mm which is incompatible with the definition of real stone free status. However, we still believe that the outcomes of the current study are valuable as, to the best of our knowledge, this is the only study in the literature focusing on URS outcomes in the elderly population.

## CONCLUSIONS

In this study, performing anterograde URS in supine position provided higher success rates, less need for ancillary procedures and similar complication rates compared to retrograde URS. Based on these results, anterograde URS shall be considered as one of the primary treatment options for management of impacted upper ureteral stones in the elderly population.

## References

[1] 1. Assimos D, Krambeck A, Miller NL, Monga M, Murad MH, Nelson CP, et al. Surgical Management of Stones: American Urological Association/Endourological Society Guideline, PART I. J Urol. 2016;196:1153-60. 10.1016/j.juro.2016.05.090 27238616

[2] 2. Türk C, Petřík A, Sarica K, Seitz C, Skolarikos A, Straub M, et al. EAU Guidelines on Interventional Treatment for Urolithiasis. Eur Urol. 2016;69:475-82. 10.1016/j.eururo.2015.07.041 26344917

[3] 3. Morgentaler A, Bridge SS, Dretler SP. Management of the impacted ureteral calculus. J Urol. 1990;143:263-6. 10.1016/s0022-5347(17)39928-7 1967657

[4] 4. Roberts WW, Cadeddu JA, Micali S, Kavoussi LR, Moore RG. Ureteral stricture formation after removal of impacted calculi. J Urol. 1998;159:723-6. 9474134

[5] 5. Gu XJ, Lu JL, Xu Y. Treatment of large impacted proximal ureteral stones: randomized comparison of minimally invasive percutaneous antegrade ureterolithotripsy versus retrograde ureterolithotripsy. World J Urol. 2013;31:1605-10. 10.1007/s00345-013-1026-2 23334470

[6] 6. Long Q, Guo J, Xu Z, Yang Y, Wang H, Zhu Y, et al. Experience of mini-percutaneous nephrolithotomy in the treatment of large impacted proximal ureteral stones. Urol Int. 2013;90:384-8. 10.1159/000343668 23635397

[7] 7. Sun X, Xia S, Lu J, Liu H, Han B, Li W. Treatment of large impacted proximal ureteral stones: randomized comparison of percutaneous antegrade ureterolithotripsy versus retrograde ureterolithotripsy. J Endourol. 2008;22:913-7. 10.1089/end.2007.0230 18429682

[8] 8. Wang Y, Zhong B, Yang X, Wang G, Hou P, Meng J. Comparison of the efficacy and safety of URSL, RPLU, and MPCNL for treatment of large upper impacted ureteral stones: a randomized controlled trial. BMC Urol. 2017;17:50. 10.1186/s12894-017-0236-0 PMC5492714 28662708

[9] 9. Yang Z, Song L, Xie D, Hu M, Peng Z, Liu T, et al. Comparative study of outcome in treating upper ureteral impacted stones using minimally invasive percutaneous nephrolithotomy with aid of patented system or transurethral ureteroscopy. Urology. 2012;80:1192-7. 10.1016/j.urology.2012.08.045 23206762

[10] 10. Zhang Y, Yu CF, Jin SH, Zhu H, Na YQ. A prospective comparative study between minimally invasive percutaneous nephrolithotomy in supine position and flexible ureteroscopy in the management of single large stone in the proximal ureter. Urology. 2014;83:999-1002. 10.1016/j.urology.2013.11.034 24507896

[11] 11. Gao ZM, Gao S, Qu HC, Li K, Li N, Liu CL, et al. Minimally invasive percutaneous nephrolithotomy improves stone-free rates for impacted proximal ureteral stones: A systematic review and meta-analysis. PLoS One. 2017;12:e0171230. 10.1371/journal.pone.0171230 PMC5289591 28152097

[12] 12. Dindo D, Demartines N, Clavien PA. Classification of surgical complications: a new proposal with evaluation in a cohort of 6336 patients and results of a survey. Ann Surg. 2004;240:205-13. 10.1097/01.sla.0000133083.54934.ae PMC1360123 15273542

[13] 13. Abdel-Khalek M, Sheir KZ, Mokhtar AA, Eraky I, Kenawy M, Bazeed M. Prediction of success rate after extracorporeal shock-wave lithotripsy of renal stones--a multivariate analysis model. Scand J Urol Nephrol. 2004;38:161-7. 10.1080/00365590310022626 15204407

[14] 14. Dhar NB, Thornton J, Karafa MT, Streem SB. A multivariate analysis of risk factors associated with subcapsular hematoma formation following electromagnetic shock wave lithotripsy. J Urol. 2004;172(6 Pt 1):2271-4. 10.1097/01.ju.0000143459.03836.2d 15538247

[15] 15. Ng CF. The effect of age on outcomes in patients undergoing treatment for renal stones. Curr Opin Urol. 2009;19:211-4. 10.1097/mou.0b013e32831e16b7 19195134

[16] 16. Prattley S, Voss J, Cheung S, Geraghty R, Jones P, Somani BK. Ureteroscopy and stone treatment in the elderly (≥70 years): prospective outcomes over 5- years with a review of literature. Int Braz J Urol. 2018;44:750-757. 10.1590/S1677-5538.IBJU.2017.0516 PMC6092651 29522293

[17] 17. Ozgor F, Yanaral F, Savun M, Ozdemir H, Caglar U, Sarilar O. Comparison of miniaturized percutaneous nephrolithotomy and flexible ureterorenoscopy for moderate size renal stones in elderly patients. Kaohsiung J Med Sci. 2018;34:352-6. 10.1016/j.kjms.2017.10.003 PMC11915566 29747780

